# Differentially Expressed MicroRNAs in Conservatively Treated Nontraumatic Osteonecrosis Compared with Healthy Controls

**DOI:** 10.1155/2018/9015758

**Published:** 2018-05-24

**Authors:** Biaofang Wei, Wei Wei, Liang Wang, Baoxiang Zhao

**Affiliations:** ^1^Department of Orthopedics, Linyi People's Hospital, Linyi, China; ^2^Department of Orthopedics, First School of Clinical Medicine, Guangzhou University of Chinese Medicine, Guangzhou, China; ^3^Department of Surgery, Shandong Medical College, Linyi, China

## Abstract

Deregulation of microRNAs (miRNAs) contributes to nontraumatic osteonecrosis of the femoral head (ONFH-N), but the differentially expressed circulating miRNAs in patients with ONFH-N receiving nonsurgical therapy are unknown. This study aimed to determine the miRNAs expression profile of patients with ONFH-N receiving conservative treatments. This was a case-control prospective study of 43 patients with ONFH-N and 43 participants without ONFH-N, enrolled from 10/2014 to 10/2016 at the Department of Orthopedics of the Linyi People's Hospital (China). The two groups were matched for age, gender, and living area. Microarray analysis and quantitative RT-PCR were used to examine the differentially expressed miRNAs. Bioinformatics was used to predict miRNA target genes and signaling pathways. Microarray and quantitative RT-PCR revealed that nine miRNAs were downregulated and five miRNAs were upregulated in ONFH-N (*n* = 3) compared with controls (*n* = 3). Bioinformatics showed that calcium-mediated signaling pathway, regulation of calcium ion transmembrane transporter activity, cytoskeletal protein binding, and caveolae macromolecular signaling complex were probably regulated by the identified differentially expressed miRNAs. In the remaining 80 subjects (*n* = 40/group), miR-335-5p was downregulated (*P* = 0.01) and miR-100-5p was upregulated (*P* = 0.02) in ONFH-N compared with controls. In conclusion, some miRNAs are differentially expressed in conservatively treated ONFH-N compared with controls. Those miRNAs could contribute to the pathogenesis of ONFH-N.

## 1. Introduction

Osteonecrosis of the femoral head (ONFH) is a common and refractory orthopedic disease. It is characterized by ischemic changes in cellular components of the femoral head (including bone, endothelial, and adipose cells) [1, 2]. Various pathogenic mechanisms of ONFH have been suggested, including metabolic disturbance of fat, damage of micrangium endothelial cells, death of osteoblasts and osteocytes, disorders of bone remodeling, decreased bone mass, and immune dysfunction [1, 2]. Nevertheless, the exact molecular mechanisms underlying nontraumatic ONFH remain poorly understood.

Traumatic and nontraumatic ONFH (ONFH-N) are not treated in the same manner and the patients have different outcomes [1, 2]. For patients in the early and middle stages, conservative treatments are always selected [1, 2]. The indications for conservative treatments are (1) patients in early-stage ischemic ONFH (because the shape of the femoral head is still adequate and the joint space is normal, using traditional Chinese medicine (TCM) and western medicine without weight bearing can effectively improve the blood circulation and restore the joint functions); (2) the health of patients ≥ 65 years of age can be less good and there is a risk of postoperative complications (if the pain is not too severe and if the hip functions are only moderately limited, using TCM can alleviate pain and improve hip functions); and (3) for patients with phase III disease and contraindication to surgery, or with bilateral ONFH, conservative treatment can be used (although conservative treatments cannot restore the shape of the femoral head, the effectiveness is still good if the treatments are appropriate and the patient is not weight bearing; after treatments, the patients can be free from pain for several years or decades) [3–5].

MicroRNAs (miRNAs) are small, endogenous, noncoding RNAs that regulate the expression of target genes through translational repression or degradation of target mRNAs. They play important roles in many biological processes such as development, differentiation, cancer, rheumatic arthritis, and bone-related diseases [6–8]. Recent studies suggested that the deregulation of miRNAs contributes to ONFH-N [9, 10]. Indeed, miR-3960 is significantly upregulated in patients with ONFH, and miR-3960 directly targets Hoxa2, a repressor of Runx2, resulting in the upregulation of bone morphogenetic protein-2- (BMP-2-) induced osteoblastogenesis [11]. miR-210 is upregulated in patients with ONFH and may be involved in ONFH pathogenesis [12]. miR-31 is upregulated in osteoclasts, where it controls osteoclast formation and bone resorption by targeting RhoA [13]. miR-30 family members negatively regulate BMP-2-induced osteoblast differentiation by directly targeting Smad1 and Runx2 [14].

Previous studies explored the global circulating miRNA expression profiles in ONFH-N [15], steroid-induced ONFH [16], and hormone-induced ONFH-N [17] and in the reparative interface of ONFH [18]. A previous study by our group demonstrated that several miRNAs (such as miR-423 and miR-10a) were dysregulated in hormone-induced ONFH-N [17]. Nevertheless, the differentially expressed circulating miRNAs in patients with ONFH-N undergoing conservative therapy are unknown.

Therefore, the aim of the present study was to identify the differentially expressed circulating miRNAs (using miRNA chip analysis) in patients with ONFH-N receiving conservative therapy. The microarray results were confirmed by real-time PCR. The bioinformatics analysis of microarray data was performed to identify the related miRNA target genes, signaling pathways, and regulatory networks.

## 2. Materials and Methods

### 2.1. Study Design and Patients

This was a case-control prospective study of 43 patients with ONFH-N and 43 participants without ONFH-N (from the hospital staff who volunteered to participate; a compensation of 100 RMB was offered) recruited between October 2014 and October 2016 at the Department of Orthopedics of the Linyi People's Hospital (China). The two groups were matched for age, gender, and living area. For the ONFH-N group, the inclusion criteria were (1) diagnosis of ONFH-N, confirmed by X-ray and magnetic resonance imaging (MRI) and according to the guidelines of the Chinese Medical Association for ONFH [19], and (2) being conservatively treated (restrained activity, TCM, and physical therapy). For both groups, the exclusion criteria were (1) any bone disease, (2) history of cancer, (3) hormone therapy, or (4) any drug known to affect bone or calcium/phosphorus metabolism.

This study was approved by the human ethics committee of Linyi People's Hospital and has been carried out in accordance with the Declaration of Helsinki. Written informed consent was obtained from each participant.

### 2.2. Data Collection

Demographic and clinical data were collected from the medical charts (patients) or by interviewing the participants (controls). Biochemical parameters were determined using routine clinical biochemistry assays.

### 2.3. RNA Extraction

Blood was collected from each participant, flash-frozen in liquid nitrogen, and stored at −80°C. Total RNA was isolated from the samples using TRIzol (Invitrogen Inc., Carlsbad, CA, USA), according to the manufacturer's protocol. RNA purity was measured using a NanoDrop 2000 spectrophotometer (Thermo Fisher Scientific, Waltham, MA, USA) and integrity was assessed by gel electrophoresis.

### 2.4. Microarray Analysis

We performed miRNA expression profiling using microarrays (Affymetrix GeneChip miRNA 4.0; Affymetrix, Santa Clara, CA, USA; containing 2578 human mature miRNA probes) on the samples from three randomly selected patients and controls (*n* = 3/group). Differentially expressed miRNAs were filtered to exclude changes <2.0-fold compared with normal controls.

### 2.5. Real-Time qPCR

The miRNAs identified using the microarray analysis were quantified by qRT-PCR in the whole cohort using the human TaqMan MicroRNA Assay Kit (Applied Biosystems, Foster City, CA, USA). The reverse transcription reaction was carried out with the TaqMan MicroRNA Reverse Transcription Kit (Applied Biosystems, Foster City, CA, USA) in 15 *μ*l containing 5 *μ*l of RNA extract, 0.15 *μ*l of 100 mM dNTPs, 1 *μ*l of MultiScribe Reverse Transcriptase, 1.5 *μ*l of 10x reverse transcription buffer, 0.19 *μ*l of RNase inhibitor, 1 *μ*l of gene-specific primer, and 4.16 *μ*l of nuclease-free water. The reaction protocol was 16°C for 30 min, at 42°C for 30 min, at 85°C for 5 min, and then held at 4°C. Then, 1.33 *μ*l of cDNA was amplified using 10 *μ*l of TaqMan 2x Universal PCR Master Mix with no AmpErase UNG (Applied Biosystems, Foster City, CA, USA), 1 *μ*l of gene-specific primers/probe, and 7.67 *μ*l of nuclease-free water in a final volume of 20 *μ*l. Quantitative PCR was run on a 7300 Real-Time PCR system (Applied Biosystems, Foster City, CA, USA). The relative expression levels of miRNAs were normalized to cel-miR-39 [20] and calculated using the 2^−ΔΔCt^ method. The expression of the miRNAs was confirmed by RT-PCR for the six patients and controls (*n* = 3/group) tested by microarray. Selected miRNAs were measured by RT-PCR among the 80 remaining subjects (*n* = 40/group).

### 2.6. Bioinformatics Analysis

To predict the target genes of the identified miRNAs, the TargetScan and miRanda databases were searched. Gene ontology (GO) analysis was applied for the functional annotation analysis of the predicted genes. Pathway analysis was completed by identifying the enriched pathways involved with these miRNAs, according to the Kyoto Encyclopedia of Genes and Genomes (KEGG).

### 2.7. Statistical Analysis

Normal distribution of the continuous variables was verified using the Kolmogorov-Smirnov test. All variables were normally distributed and presented as mean ± standard deviation (SD). Comparisons between groups were performed using Student's *t*-test. All statistical analyses were conducted using SPSS 18.0 (IBM, Armonk, NY, USA). Fisher's exact test and the chi-square test were used for the GO categories and for the selection of the significant pathways. Two-sided *P* values < 0.05 were considered statistically significant.

## 3. Results

### 3.1. Characteristics of the Subjects

There were no differences between two groups regarding age and gender (Table 1). The serum leptin and ApoB levels were higher in ONFH patients compared with controls (both *P* < 0.001), while serum adiponectin and ApoA1 levels were lower (both *P* < 0.001).

### 3.2. miRNA Expression Profiles

**Figure 1:**
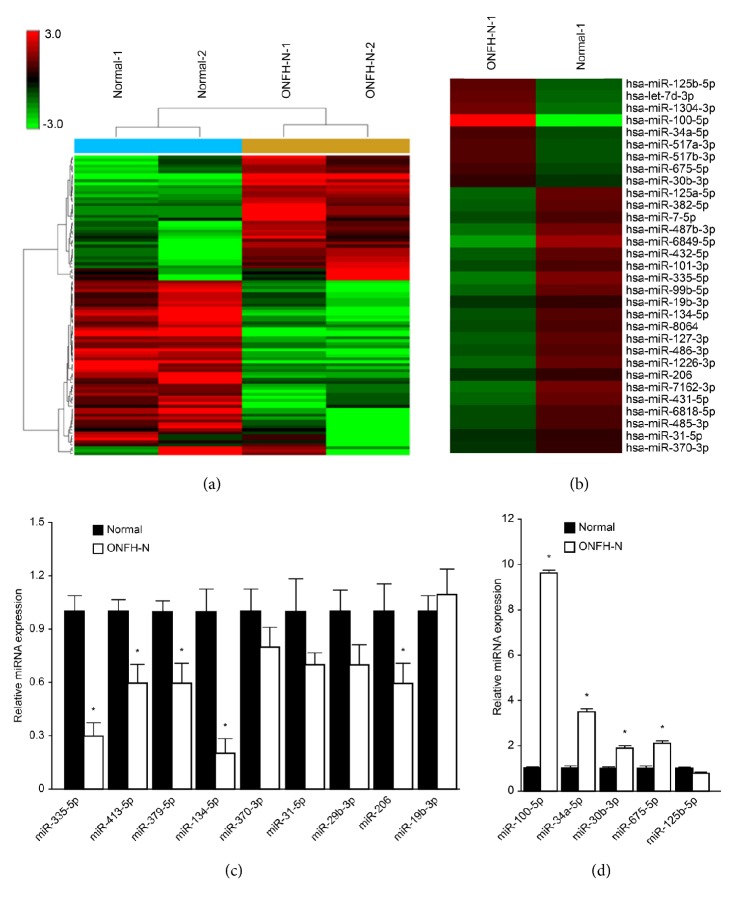
*miRNA profiles between patients with nontraumatic ONHF and controls*. (a-b) Hierarchical clustering for the differentially expressed miRNAs (^*∗*^*P* < 0.05, Student's *t*-test; *n* = 3/group; only one control and one patient are shown). (c-d) Validation of miRNA microarray results by real-time qPCR (*n* = 3/group).

A total of 102 miRNAs demonstrated a ≥2.0-fold change in expression and were analyzed using hierarchical clustering to generate a heatmap (Figure 1(a)) using three patients and three controls. Of the 102 differentially expressed miRNAs, 31 were related to bone metabolism and bone remodeling (Figure 1(b)).

To validate the microarray results, nine downregulated (miR-335-5p, miR-413-5p, miR-379-5p, miR-134-5p, miR-370-3p, miR-31-5p, miR-29b-3p, miR-206, and miR-19b-3p) and five upregulated (miR-100-5p, miR-34a-5p, miR-30b-3p, miR-675-5p, and miR-125b-5p) miRNAs were selected for real-time PCR analysis using the serum samples used in the microarray assay (*n* = 3/group). Similar results were obtained, as shown in Figures 1(c) and 1(d). As shown in Figure 1(c), the expression of miR-335-5p and miR-134-5p was lower in ONFH-N compared to normal healthy controls. Significant changes in the expression of miR-413-5p, miR-379-5p, and miR-206 were observed. There was no significant difference in miR-370-3p, miR-31-5p, miR-29b-3p, and miR-19b-3p between ONFH-N and controls. We observed higher expression of miR-100-5p, miR-34a-5p, miR-30b-3p, and miR-675-5p in ONFH-N patients compared with healthy controls (Figure 1(d)).

### 3.3. Bioinformatics Analysis

**Figure 2:**
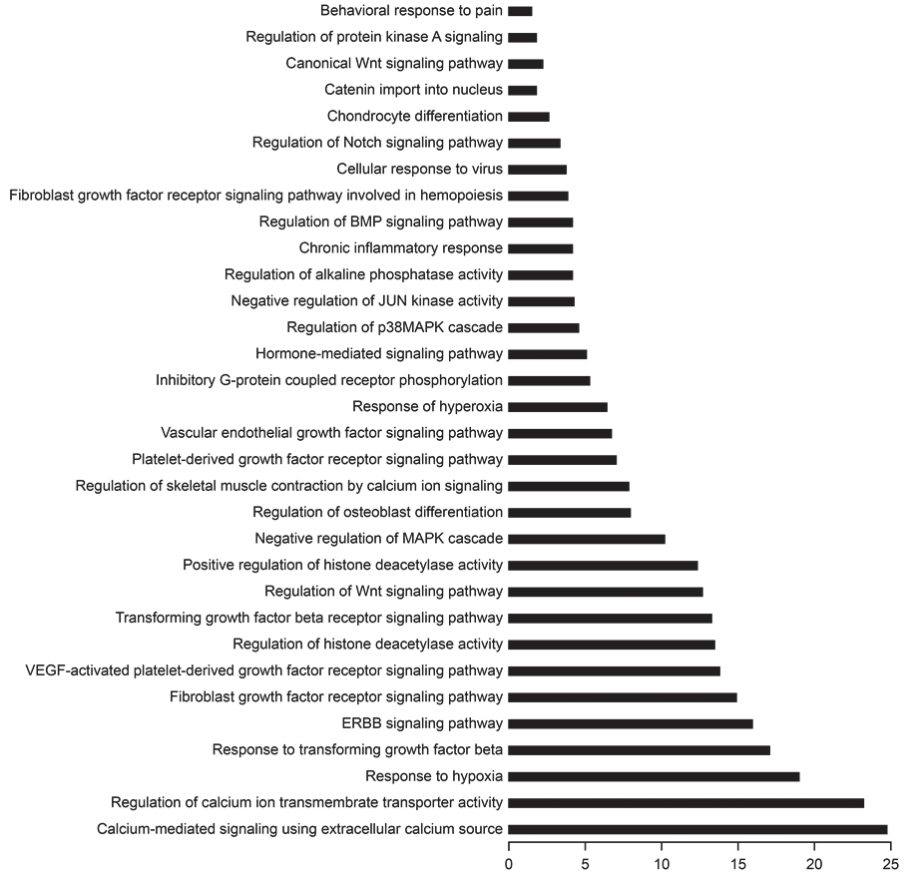
*GO category and KEGG analysis*. The calcium-mediated signaling pathway was the most enriched signaling pathway, and regulation of calcium ion transmembrane transporter activity was the most significantly enriched biological process among GO categories.

**Figure 3:**
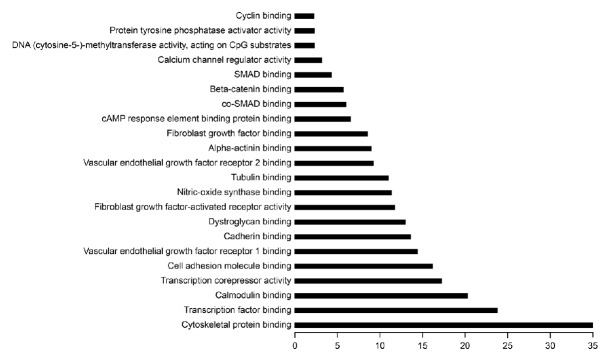
*Partial GO enrichment for the predicted target genes among molecular functions*. Cytoskeletal protein binding and caveolae macromolecular signaling complex were the most significantly enriched molecular functions.

**Figure 4:**
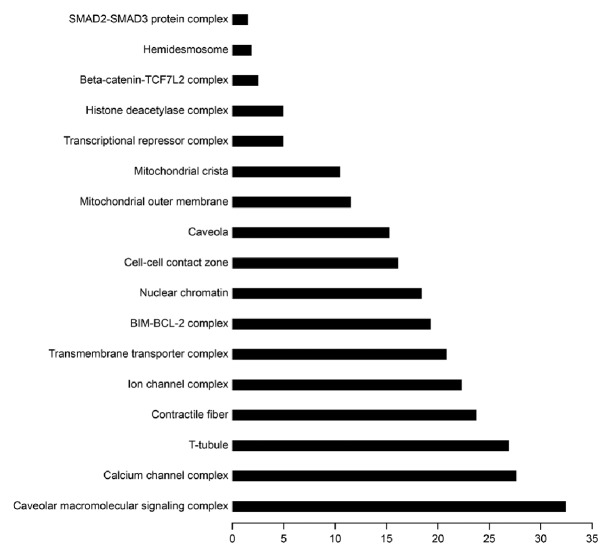
*Partial GO enrichment for the predicted target genes among cellular components*. Cellular components were significantly enriched.

To better understand the possible functions of the differentially expressed miRNAs, we performed GO and KEGG analyses [21]. As shown in Figure 2, the calcium-mediated signaling pathway was the most enriched one, and regulation of calcium ion transmembrane transporter activity was the most significantly enriched biological process among GO categories. Moreover, cytoskeletal protein binding and caveolae macromolecular signaling complex were the most significantly enriched molecular functions (Figure 3) and cellular components (Figure 4) among GO categories, respectively.

### 3.4. Expression of miR-335-5p and miR-100-5p

**Figure 5:**
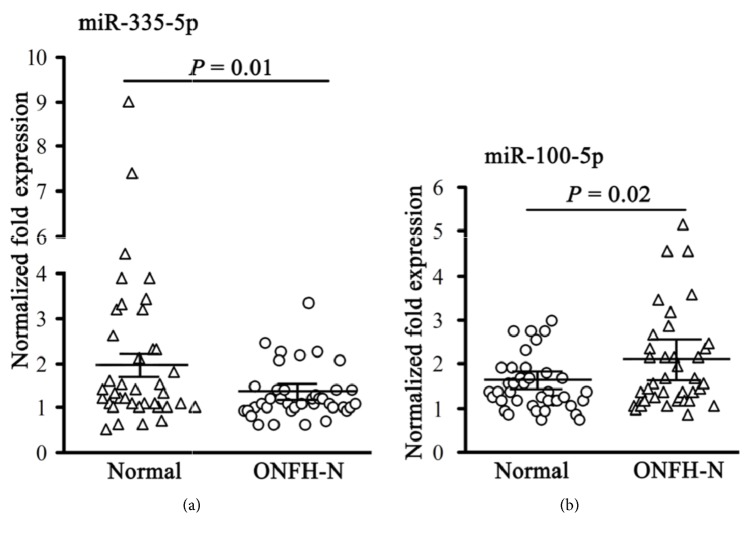
Expression of miR-335-5p and miR-100-5p in 80 samples (*n* = 40/group).

To confirm the miR-335-5p and miR-100-5p expression profiles, RT-PCR was performed in the remaining 80 samples (*n* = 40/group) that were not used for the microarray assay. In line with the data above, miR-335-5p was downregulated in patients with nontraumatic ONFH compared with controls (*P* = 0.01; Figure 5(a)). In contrast, the expression of miR-100-5p in patients with nontraumatic ONFH was higher than in controls (*P* = 0.02; Figure 5(b)).

## 4. Discussion

Deregulation of miRNAs contributes to ONFH-N [22], but the differentially expressed circulating miRNAs in patients with ONFH-N receiving conservative therapy are unknown. Therefore, this study aimed to determine the differentially expressed miRNAs in patients with ONFH-N receiving conservative treatments. This study revealed differentially expressed miRNA between ONFH-N and controls. These miRNAs might contribute to the pathogenesis of ONFH-N.

ONFH is a devastating disease that eventually leads to the collapse of the femoral head and the need for hip replacement [23, 24]. ONFH is characterized by imbalance between the catabolic and anabolic actions of the osteoclasts [25]. miRNAs are regarded as common regulators in the biological and pathological processes of a variety of diseases. Previous studies revealed dysregulated miRNAs in osteosarcomas, osteogenesis imperfecta, and osteoporotic fractures [26–28]. Therefore, miRNA could be used as biomarkers of bone tissue disorders.

In the present study, using microarray analysis, 31 miRNAs were significantly differentially expressed between ONFH-N and controls. Real-time PCR revealed that nine miRNAs (miR-335-5p, miR-413-5p, miR-379-5p, miR-134-5p, miR-370-3p, miR-31-5p, miR-29b-3p, miR-206, and miR-19b-3p) were downregulated and five miRNAs (miR-100-5p, miR-34a-5p, miR-30b-3p, miR-675-5p, and miR-125b-5p) were upregulated in patients with ONFH-N compared with controls, suggesting that these miRNAs could be involved in ONFH-N. In addition, bioinformatics analysis showed that calcium-mediated signaling pathway, regulation of calcium ion transmembrane transporter activity, cytoskeletal protein binding, and caveolae macromolecular signaling complex were most likely to be regulated by these differentially expressed miRNAs. A previous study identified 39 miRNAs that were differentially regulated between ONFH-N and controls, including miR-355-5p [15], supporting the present study, but discrepancies between the two studies might be due to a number of factors, including genetics and environment.

The role of miR-335-5p on Wnt signaling has been described in various cancers [29, 30]. Previous observations suggested that upregulation of miR-335 could be a critical regulatory event in the biology of mesenchymal stem cells (MSCs), impairing the MSC reparative phenotype [31]. In the initial stages of osteogenesis, miR-335-5p downregulates the Wnt antagonist DKK1 [32]. Furthermore, miR-335-5p is downregulated in osteoarthritis MSCs compared to control MSCs [33], supporting the results of the present study, i.e., that decreased expression of miR-335-5p in ONFH-N could promote osteonecrosis. Indeed, downregulation of miR-335-5p has been reported to be associated with ONFH, suggesting that it could be a key mediator of ONFH [15]. Conservative treatments of ONFH-N might not affect miR-335-5p expression since the present study also showed low expression of miR-355-5p despite treatments. Indeed, according to previous reports, the expression of miR-335-5p was significantly decreased in ONFH patients [15, 31–33]. Taken together, these results suggest that the lower expression of miR-335-5p could contribute to ONFH. Generally speaking, if a treatment could increase the expression of miR-335-5p, this treatment could be appropriate for ONFH therapy, but such treatment has not been demonstrated yet. Therefore, the outcomes from conservative treatments might not be mediated by miR-335-5p.

Recent studies showed that miR-100 was aberrantly expressed in different types of cancers [34–37] and could be a tumor suppressor. Zeng et al. [38] reported that miR-100 regulates the osteogenic differentiation of human adipose-derived MSCs by targeting BMPR2, and the expression of miR-100 inhibits the osteogenic differentiation of human adipose-derived mesenchymal stem cells. In the present study, miR-100 expression was upregulated in ONFH-N compared to controls. Precise data about the roles of miR-100 in bone metabolism are lacking, but these data might suggest that higher expression of miR-100 in ONFH-N is associated with deregulated bone metabolism and impaired osteogenic differentiation, but additional studies are necessary to examine this issue.

ONFH is a common bone disease, especially in the elderly population. Without proper treatment, the patients may progress to complete collapse of the femoral head. Conservative treatments of ONFH-N have obvious advantages. For instance, there is no surgical trauma and no need for hospitalization, and medical costs are low. Nevertheless, it is important to evaluate adequately the effects of conservative treatments on ONFH-N. Accumulating evidence revealed the critical role of miRNAs in bone metabolic diseases. Different miRNA expression profiles in nontraumatic ONFH patients were explored [15–18], and these findings may result in novel potential biomarkers for the early diagnosis of ONFH. In this study, we focused on the different expression levels of miRNAs between ONFH patients receiving conservative treatments and healthy normal controls. The results may suggest a novel strategy to help estimate the therapeutic effect of nonsurgical treatment and prognosis. Furthermore, identifying the differentially expressed miRNAs between these two groups may also help to better understand the underlying mechanisms of ONFH.

The present study has limitations. First, although many dysregulated miRNAs were identified, the targets of those miRNAs are still unknown. miRNAs regulate protein expression of target genes through translation repression or degradation of target mRNAs. Nevertheless, bone tissue specimens were not obtained, especially from healthy volunteers, which would be unethical. Secondly, the sample size was small and this may influence the total miRNA expression profiles. In addition, the patients were at stage IIIB/IIIC, making them surgical candidates, but surgery was kept for later either because the symptoms were still manageable or because the patients refused surgery. Thirdly, not all identified miRNAs could be tested by RT-PCR because of the costs involved. Therefore, two miRNAs that had a possible association with bone metabolism were selected [31, 33, 38]. Finally, as shown in Table 1, the levels of leptin and ApoB were higher in patients with ONFH-N than in controls, while the concentrations of ApoA1 and adiponectin were lower. Previous studies reported that the factors involved in lipid metabolism (such as leptin, ApoB, ApoA1, and adiponectin) may be correlated with ONFH [39, 40], but little is known about the roles of these factors in the pathogenesis and progression of ONFH. Additional studies are needed to reveal the functions of these factors in ONFH-N. In the future, it will be interesting to study whether there are influence and correlations between the types, duration, and frequency of conservative treatments and miRNA expression levels. In addition, comparisons could be made with patients who underwent surgery.

## 5. Conclusions

In conclusion, this study revealed differentially expressed miRNA in ONFH-N under conservative treatments. Bioinformatics could be a useful tool to predict the signaling pathways or mechanisms of these miRNAs. Additional investigations are needed to identify the exact involvement of these miRNAs in the pathogenesis of ONFH.

## Figures and Tables

**Table 1:** Characteristics of the subjects.

	Patients (*n* = 43)	Controls (*n* = 43)	*P*
Male/female	28/15	25/18	0.66
Age	55.2 ± 6.9	53.3 ± 8.7	0.27
Chinese ONFH classification			- - -
III B	19	- - -	
III C	24	- - -	
Leptin	15.7 ± 4.0^*∗*^	11.6 ± 3.6	<0.001
ApoA1 (g/L)	0.96 ± 0.24^*∗*^	1.53 ± 0.34	<0.001
ApoB (g/L)	1.04 ± 0.27^*∗*^	0.74 ± 0.19	<0.001
Adiponectin (mg/L)	7.6 ± 1.9^*∗*^	10.3 ± 2.2	<0.001

^*∗*^ *p* < 0.001 versus controls.
